# Nucleotide-Induced Conformational Changes in *Escherichia coli* DnaA Protein Are Required for Bacterial ORC to Pre-RC Conversion at the Chromosomal Origin

**DOI:** 10.3390/ijms161126064

**Published:** 2015-11-24

**Authors:** Rahul Saxena, Sona Vasudevan, Digvijay Patil, Norah Ashoura, Julia E. Grimwade, Elliott Crooke

**Affiliations:** 1 Department of Biochemistry and Molecular & Cellular Biology Georgetown University Medical Center, Washington, DC 20007, USA; sv67@georgetown.edu (S.V.); dap89@georgetown.edu (D.P.); crooke@georgetown.edu (E.C.); 2 Department of Biological Sciences, Florida Institute of Technology, 150 West University Blvd, Melbourne, FL 32901, USA; nashoura2010@utexas.edu (N.A.); grimwade@fit.edu (J.E.G.); 3 Lombardi Comprehensive Cancer Center, Georgetown University Medical Center, Washington, DC 20007, USA

**Keywords:** DnaA protein, AAA+ domain, molecular docking, proteolysis, chromosomal origin, DNA replication

## Abstract

DnaA oligomerizes when bound to origins of chromosomal replication. Structural analysis of a truncated form of DnaA from *Aquifex aeolicus* has provided insight into crucial conformational differences within the AAA+ domain that are specific to the ATP- *versus* ADP- bound form of DnaA. In this study molecular docking of ATP and ADP onto *Escherichia coli* DnaA, modeled on the crystal structure of *Aquifex aeolicus* DnaA, reveals changes in the orientation of amino acid residues within or near the vicinity of the nucleotide-binding pocket. Upon limited proteolysis with trypsin or chymotrypsin ADP-DnaA, but not ATP-DnaA generated relatively stable proteolytic fragments of various sizes. Examined sites of limited protease susceptibility that differ between ATP-DnaA and ADP-DnaA largely reside in the amino terminal half of DnaA. The concentration of adenine nucleotide needed to induce conformational changes, as detected by these protease susceptibilities of DnaA, coincides with the conversion of an inactive bacterial origin recognition complex (bORC) to a replication efficient pre-replication complex (pre-RC) at the *E. coli* chromosomal origin of replication (*oriC*).

## 1. Introduction

Initiation of *Escherichia coli* chromosomal replication at a unique 245 base pair origin of replication (*oriC*) is mediated by DnaA protein [1,2]. DnaA contains four distinct domains (domain I–IV), each of which has been assigned with distinct function(s) [3,4]. Amino terminal residues 1–86 [3,4] that comprise domain I are involved in DnaA-DnaA interaction [5,6], interactions with DiaA [7], and the loading of DnaB helicase [6,8,9]. Site-directed mutagenesis [10] as well as nuclear magnetic resonance (NMR) structural analysis [8] of domain I revealed the importance of Trp6 for DnaA protomer-protomer interaction and the role of Glu-21 in providing the surface for DnaB helicase loading. Domain I is followed by a flexible linker, domain II, that spans between amino acid residues 87–134 [3,4]. The function of domain II has been suggested to properly align domain I with domains III–IV [3,4]. Deletion analyses within domain II identified regions that are dispensable and do not adversely affect the viability of *E. coli* [11]. This study also suggested different boundaries for domain II, spanning amino acid residues 79–135 [11]. However, recently, nonessential regions present in domain II have been proposed to play a role in replication initiation by promoting the recruitment of DnaB to *E. coli oriC* [12].

DnaA domain III (amino acid residues 135–356), like other AAA+ (ATPase associated with cellular activities) protein family members, contains a common core structure [13,14] of α-β-α motifs (α1–α11 and β1–β5 of domain III) that combine to form a nucleotide-binding fold for DnaA protein [15,16]. Beside this, other salient features of domain III are the presence of Walker A (also known as Walker loop or P loop) and Walker B motifs that contain the consensus sequence GXXXXGK (T/S) and hhhhDE, respectively [13,14,15,16]. A high-resolution X-ray crystal structure of a truncated (domain III–IV) DnaA protein from the thermophilic bacterium *Aquifex aeolicus* revealed a bipartite ATP-binding site formed at the interface of neighboring DnaA protomers, involving interaction between Walker A and Walker B sequence motifs of one DnaA with Arg-334 of the other monomer [16]. Amino acid residue Lys-178 present in Walker A motif and Asp-235 present in Walker B motif are involved in high affinity adenine nucleotide binding and subsequent opening of the DNA duplex [17]. In addition, other amino acid residues, such as Ala-184 [18,19], Trp-271 [19] and Asp-269 [20] are present in the nucleotide binding domain III and implicated in nucleotide binding.

Lastly, domain IV (amino acids 375–467) has a helix-loop-helix motif (α1–α5 of domain IV), required for DNA binding and the subsequent formation of nucleoprotein complexes at *oriC* [21,22]. Moreover, a small amphipathic helix (containing amino acid residues 357–374) spanning domains III and IV is required for the peripheral association of DnaA with acidic lipid bilayers [23,24].

*E. coli oriC* contains specific DnaA recognition sites termed as high affinity sites- R1, R2 and R4 [25,26,27,28,29,30,31] and low affinity sites-I1, I2, I3, R5M, τ1, τ2 and C1, C2, & C3 [26,27,28,29,30,31]. ADP-DnaA can occupy high-affinity DnaA sites [26,28,29,30,31], as well as R5M and C1 [32,33], to generate bacterial origin replication complexes, or bORC. In contrast, ATP-DnaA binds the low-affinity DnaA recognition sequences in *oriC* significantly better than ADP and is required to form replication-efficient pre-replication complexes, or pre-RC [26,27,28,29,30,31,32,33]. The differing abilities of ADP-DnaA and ATP-DnaA to multimerize on *oriC* sites argue for different conformations between the two nucleotide forms of DnaA. The previously reported crystal structure of truncated *A. aeolicus* DnaA protein highlights major conformational differences between the two nucleotide forms of DnaA protein [15,16]. The non-hydrolyzing ATP analogue, AMP-CPP bound to truncated *A. aeolicus* DnaA protein induces specific conformational changes promoting the formation of filamentous or helical structures that stabilize protein subunit-subunit interactions. These conformational changes are not sterically compatible for ADP-DnaA [16]. Moreover, another study reported that the presence of ATP, AMPPNP and ATPγS strongly induce the generation of higher-order DnaA structures [34].

Amino acid residues present in *A. aeolicus* DnaA (corresponding *E. coli* DnaA residues in parentheses), such as Arg-277 (Arg-334), Glu-280 (Glu-337), Arg-230 (Arg-285), Ser-229 (Ser-284), are required for both ATP binding and DnaA-DnaA assembly [14,29,35]. While *A. aeolicus* and *E. coli* DnaA have 65% similarity and 35% amino acid identity [14], incomplete structural data about the latter limits our knowledge of important conformational changes likely to be present between *E. coli* ADP-DnaA and ATP-DnaA. In this study molecular docking of ADP and ATP onto *E. coli* DnaA modeled on the *A. aeolicus* crystal structure, suggested that the two nucleotides interact differently with amino acid residues surrounding the nucleotide-binding pocket of *E. coli* DnaA. Interestingly, DnaA protein in the presence of ATP or ADP at amounts similar to their cellular concentrations resulted in different degrees of resistance to trypsin or chymotrypsin proteases. Alignment of the differential proteolytic fragments with full length *E. coli* DnaA facilitated identification of regions within DnaA that had conformational differences induced by ATP *versus* ADP. Our study also revealed that the conformational changes that induced efficient pre-RC assembly required comparable levels of ATP, similar to those found *in vivo*.

## 2. Results

### 2.1. Conformations of Bound ATP and ADP to DnaA

**Figure 1:**
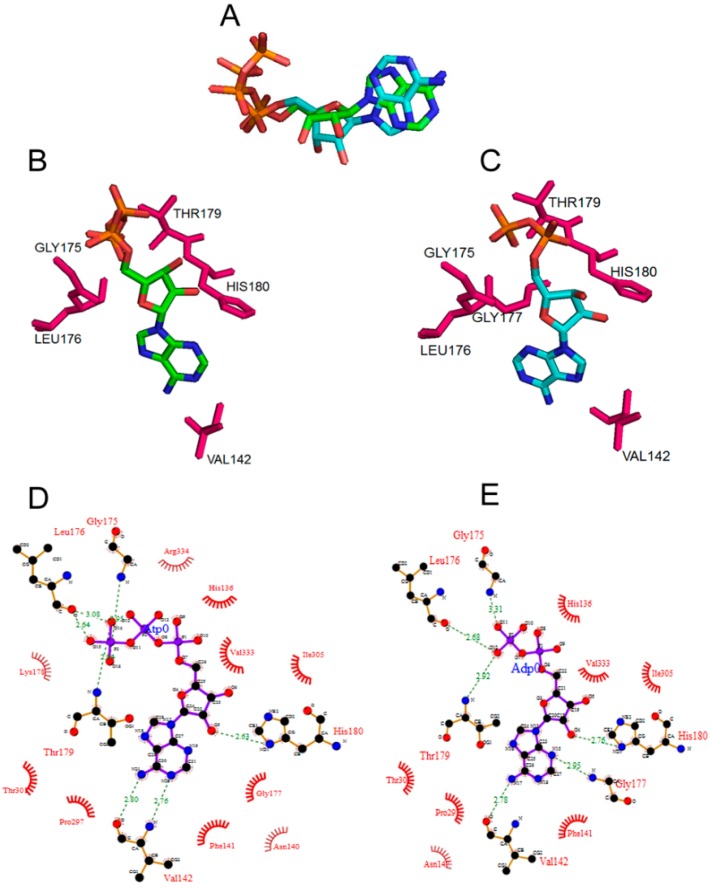
The amino acid residues forming the nucleotide-binding pocket of DnaA protein bound to ADP and ATP adopt different confirmations. (**A**) Overlap of the conformations of ADP and ATP present in the modeled *E. coli* DnaA protein in their bound state; (**B**,**C**) Amino acid residues interacting with the ATP and ADP. Figures were generated using PYMOL software; (**D**,**E**) Molecular interactions of residues in the nucleotide binding pocket with bound ATP (**D**) or ADP (**E**), hydrogen bond interactions are indicated as dashed lines in green and the van der Waals interaction are indicated as half-circles in red wire diagrams. The numbers indicate the hydrogen bonding distance between the atoms in Å units. The Figures were generated using PDBSum.

A structure-guided homology model of full-length *E. coli* DnaA docked with ATP and ADP was built based on known structures of bacterial homologs [6,15,16]. Our model suggests that while the overall fold of DnaA protein, whether bound to ATP and ADP, remains essentially the same, with an overall root mean square deviation (RMSD) of about 1 Å, there are significant local rearrangements of the binding pocket residues. Furthermore, super-positioning of the docked ATP and ADP to DnaA (using all atoms) revealed an RMSD of 2.275 Å (Figure 1A). DnaA binding of ATP and ADP occurs within the binding pocket formed by residues Val-142, Gly-175, Leu-176, Thr-179 and His-180 for ATP (Figure 1B) [15], and in addition, Gly-177 for ADP (Figure 1C). DnaA binding of ATP and ADP is stabilized by H-bonds. However, there is a predicted re-arrangement of amino acids in the binding pocket to accommodate the more bulky ATP (Figure 1D,E). The orientations and the hydrogen bonding patterns between the two nucleotide forms are notably different (Figure 1D,E). For example Gly-175 and Gly-177 both form hydrogen bonds with ADP (Gly-175 with the β-phosphate and Gly-177 with the nitrogen atom of the adenine ring). Contrary to that only Gly-175 forms a hydrogen bond with the γ-phosphate of ATP (Figure 1D,E). Similarly, Val-142 forms two hydrogen bonds with ATP, but only one with ADP. Furthermore, we also noticed the re-arrangement of residues that are at van der Waals distance (Figure 1D,E).

### 2.2. ATP-DnaA and ADP-DnaA Have Different Susceptibilities to Proteases

**Figure 2:**
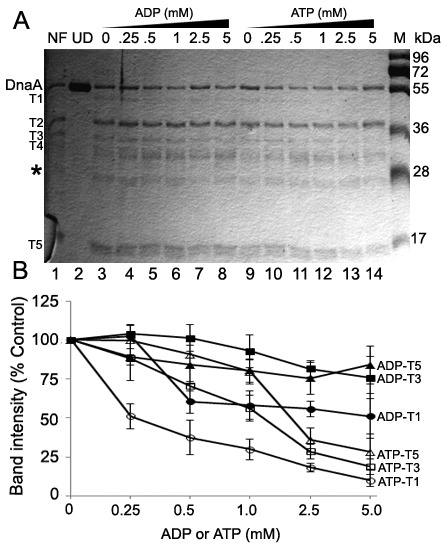
ATP-DnaA and ADP-DnaA have different susceptibility to trypsin proteolysis. (**A**) Nucleotide free, ADP and ATP-DnaA protein (1.5 μM) were subjected to proteolysis by trypsin (protein: protease molar ratio of 4.0) in the presence of additional ADP and ATP for 30 min at 30 °C. *Lane* 1, Nucleotide free DnaA (NF) treated with trypsin; *Lane* 2, undigested (UD) nucleotide free DnaA; *Lane* 3, ADP-DnaA treated with trypsin; *Lanes* 4–8, ADP-DnaA treated with trypsin in the presence of 0.25, 0.5, 1.0, 2.5 and 5 mM ADP; *Lane* 9, ATP-DnaA treated with trypsin; *Lanes* 10–14, ATP-DnaA treated with trypsin in the presence of 0.25, 0.5, 1.0, 2.5 and 5 mM ATP; (**B**) A value of 100 corresponds to the intensity of the Fragments T1, T3, T5 present in the ADP-DnaA and ATP-DnaA (*Lanes* 3 and 9, *i.e.*, no additional ADP or ATP in the reactions) respective to the band intensities present at each nucleotides concentration. Asterisk (*) indicates the respective position of the band for trypsin and the numbers denote the molecular weight in kDa. Densitometry values for band intensity corresponding to each proteolytic fragment were calculated using an identical size area minus a same size area in a protein free background from part of the gel. Data shown here is the mean (±SD) from three independent experiments. The SDS-PAGE from one of the experiment is shown.

**Figure 3:**
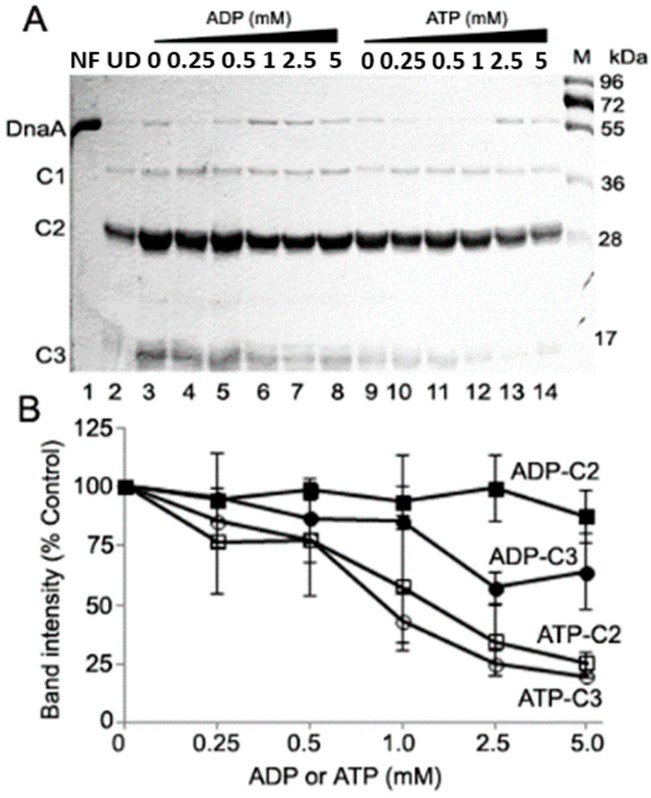
ADP-DnaA and ATP-DnaA confers different resistance to chymotrypsin protease. (**A**) Nucleotide free, ADP and ATP-DnaA protein (1.5 μM) were subjected to proteolysis by chymotrypsin (protein: protease molar ratio of 4.0) for 30 min at 30 °C. *Lane* 1, Undigested (UD) nucleotide free DnaA; *Lane* 2, Nucleotide free DnaA (NF) treated with chymotrypsin; *Lane* 3, ADP-DnaA treated with chymotrypsin; *Lanes* 4–8, ADP-DnaA treated with chymotrypsin in the presence of 0.25, 0.5, 1.0, 2.5 and 5 mM ADP; *Lane* 9, ATP-DnaA treated with chymotrypsin; *Lanes* 10–14, ATP-DnaA treated with chymotrypsin in the presence of 0.25, 0.5, 1.0, 2.5 and 5 mM ATP; (**B**) A value of 100 corresponds to the intensity of the Fragment C2 and C3 present in the ADP-DnaA and ATP-DnaA (*Lanes* 3 and 9, *i.e.*, no additional ADP or ATP in the reactions) respective to the band intensities present at each nucleotides concentration. Numbers denote the molecular weight in kDa. The values for band intensity corresponding to each proteolytic fragment were calculated by selecting a similar size area minus identical background from an empty part of the gel. Data shown here is the mean (±SD) from three independent experiments, including values from the representative SDS-PAGE.

To test for the above-predicted local rearrangements, we examined the susceptibility of ATP-DnaA *versus* ADP-DnaA to limited digestion by proteases at a DnaA-to-protease molar ratio of 4.0. Briefly, ADP-DnaA and ATP-DnaA were incubated in the absence or presence of various amounts of their respective adenine nucleotides and digested with trypsin or chymotrypsin. Resulting fragments were resolved by SDS-PAGE and visualized by Coomassie staining (Figure 2 and Figure 3).

Treatment of nucleotide-free DnaA (NF), ADP-DnaA and ATP-DnaA with trypsin generated a mix of proteolytic fragments, including T1 (~46 kDa), T3 (~33 kDa) and T5 (~20 kDa) (Figure 2A,B *cf.* (Compare *Lanes*) *Lanes* 1, 3 & 9). Immunoblotting with anti-DnaA antiserum confirmed that the proteolytic fragments were derived from DnaA protein (Figure S1). Inclusion of 0.25 mM ADP did not cause significant changes in the recovery of Fragments, T1, T3 and T5 (Figure 2A,B, *Lane* 4). Whereas addition of 0.5 mM ADP did not significantly affect band intensity of T3 or T5, less Fragment T1 was generated at this concentration of ADP (Figure 2A,B, *Lane* 5). Further, increases in the concentrations of ADP to 1, 2.5 and 5 mM resulted in subtle decreases in the recovery of Fragments T3 (from 0.9-fold to 0.8-fold to 0.75-fold, respectively), T5 (from 0.8-fold that further remains constant) and a slightly larger decrease in the band intensities of Fragment T1 (from 0.58-fold to 0.55-fold to 0.51-fold respectively) (Figure 2A,B, *cf. Lanes* 6–8).

Similar to ADP, the addition of 0.25 mM ATP did not result in any noticeable differences in the recovery of Fragments, T3 and T5 (Figure 2A,B, *cf. Lanes* 9 and 10). However, 0.25 mM ATP resulted in a relatively more pronounced decrease in the recovery of Fragment T1 (0.51-fold) (Figure 2,B, *cf. Lanes* 9 and 10). Further addition of 0.5, 1, 2.5 and 5 mM ATP resulted in the more drastic reductions in the band intensities of Fragments T3 (from 0.7-fold to 0.56-fold to 0.28-fold to 0.18-fold, respectively), T5 (0.9-fold to 0.79-fold to 0.35-fold to 0.28-fold, respectively) and Fragment T1 (0.37-fold to 0.29-fold to 0.18-fold to 0.09-fold, respectively) (Figure 2A,B, *cf. Lanes* 11–14). An earlier study [20] revealed no differences in the proteolytic patterns of ADP-DnaA *versus* ATP-DnaA with inclusion of additional respective nucleotide when treated with trypsin at a protein to protease molar ratio of 1.0. Using a similar ratio, we also did not see any differences in proteolytic cleavage between the nucleotide forms of DnaA (data not shown).

Treatment of NF, ADP-DnaA and ATP-DnaA with chymotrypsin produced a mixture of proteolytic fragments that were resolved by SDS-PAGE, including, C2 (~29.0 kDa) and C3 (~19 kDa) (Figure 3A,B *cf. Lanes* 2, 3 & 9). The origin of the proteolytic fragments was confirmed by immunoblotting with anti-DnaA antiserum (Figure S2). Inclusion of 0.25, 0.5, 1.0, 2.5 and 5 mM ADP resulted in no, to a marginal decrease in the recovery of Fragment C2, and a gradual decrease in the band intensity of Fragment C3 (from 0.95-fold to 0.86-fold to 0.85-fold to 0.6-fold that further remained constant, respectively) (Figure 3A,B, *cf. Lanes* 4–8). Similar to ADP, inclusion of 0.25 or 0.5 mM ATP resulted in a marginal decrease in the recovery of Fragments C2 (band intensity ~77-fold) and C3 (band intensity from 0.85-fold to 0.77-fold) (Figure 3A,B, *cf. Lanes* 10 and 11). On the contrary, inclusion of additional amounts of ATP to 1, 2.5 and 5 mM ATP resulted in a relatively more pronounced decrease in the band intensities of C2 (from 0.57-fold to 0.34-fold to 0.25-fold) and C3 (0.43-fold to 0.25-fold to 0.19-fold). (Figure 3A,B, *cf. Lanes* 12–14).

The inability to detect a band of chymotrypsin at the expected position on SDS-PAGE is likely due to overlapping migration of chymotrypsin and Fragment C2, considering their similar molecular weight as well as the significantly lower amount of protease (~nine folds) compared to the amount of DnaA protein loaded per sample. Combined, the results from proteolytic cleavage analyses (Figure 2 and Figure 3) suggest that levels of ATP higher than that needed to form a nucleotide-bound form, but approximating that found in cells, alter the conformation of DnaA protein.

### 2.3. Proteolytic Susceptibility Sites Predicts Structural Differences between ATP-and ADP-DnaA in Domains II and III

Untreated DnaA protein (Figure 2A, *Lane* 2) and proteolytic fragments of interest obtained following treatment with trypsin (T1, T3, T4, T5 Figure 2 *Lane* 3), or chymotrypsin (C2 and C3 Figure 3 *Lane* 3) were subjected to in-gel tryptic digestion. LCMS/MS analysis of full length DnaA and Fragments T1, T3, T4, T5, C2 & C3 (Table 1) was used to perform SWISS-PROT database searches in order to confirm the identity of each proteolytic fragment.

**Table 1:** Differential proteolytic cleavages sites between ADP-DnaA and ATP-DnaA reside in domains I, II and III. Peptide sequences of the in-gel trypsin digested fragments (T1, T3, T4, T5 and C2, C3) obtained by LCMS with ≥95% confidence level were searched against SWISS-PROT databases using ProteinPilot software 4.0 and Paragon Algorithm. The numbers flanking the peptide sequences denotes the first and the last amino acid residues of each peptide obtained in LCMS analysis after in-gel Trypsinogenesis. The number written adjacent to each proteolytic fragment indicates its size in kDa. [X] indicate the peptide sequence present in the respective fragments; and [–] stands for blank units.

No.	Peptide Sequence (in Kilo-Daltons)	T1 (45.9)	T3 (32.6)	T4 (30.4)	T5 (19.8)	C2 (29.0)	C3 (18.7)
1	^13^LQDELPATEFSMWIRPLQAELSDNTLALYAPNR^45^	-	-	-	X	-	-
2	^46^FVLDWVR^52^	X	-	-	X	-	-
3	^76^FEVGTKPVTQTPQAAVTSNVAAPAQVAQTQPQR^108^	-	-	-	X	-	X
4	^115^SGWDNVPAPAEPTYR^129^	X	-	-	-	X	X
5	^136^HTFDNFVEGK^145^	-	X	-	X	X	-
6	^156^QVADNPGGAYNPLFLYGGTGLGKTHLLHAVGNGIMAR^192^	X	X	X	X	X	-
7	^213^ALQNNAIEEFKR^224^	X	X	X	-	X	-
8	^228^SVDALLIDDIQFFANKERSQEEFFHTFNALLEGNQQIILTSDR^273^	X	X	X	-	X	X
9	^286^FGWGLTVAIEPPELETR^302^	X	X	X	-	X	X
10	^309^KADENDIRLPGEVAFFIAKR^328^	X	X	X	-	X	-
11	^335^ELEGALNRVIANANFTGRAITIDFVR^381^	X	X	X	-	X	-
12	^365^DLLALQEKLVTIDNIQK^381^	X	X	X	-	X	-
13	^416^ELTNHSLPEIGDAFGGR^432^	X	X	X	-	-	-
14	^448^EESHDIKEDFSNLIR^463^	X	-	-	-	-	-

DnaA: ^1^MSLSLWQQCLARLQDELPATEFSMWIRPLQAELSDNTLALYAPNRFVLDWVRDKYLNNINGLLTSFCGADAPQLRFEVGTKPVTQTPQAAVTSNVAAPAQVAQTQPQRAAPSTRSGWDNVPAPAEPTYRSNVNVKHTFDNFVEGKSNQLARAAARQVADNPGGAYNPLFLYGGTGLGKTHLLHAVGNGIMARKPNAKVVYMHSERFVQDMVKALQNNAIEEFKRYYRSVDALLIDDIQFFANKERSQEEFFHTFNALLEGNQQIILTSDRYPKEINGVEDRLKSRFGWGLTVAIEPPELETRVAILMKKADENDIRLPGEVAFFIAKRLRSNVRELEGALNRVIANANFTGRAITIDFVREALRDLLALQEKLVTIDNIQKTVAEYYKIKVADLLSKRRSRSVARPRQMAMALAKELTNHSLPEIGDAFGGRDHTTVLHACRKIEQLREESHDIKEDFSNLIRTLSS^467^.

**Figure 4:**
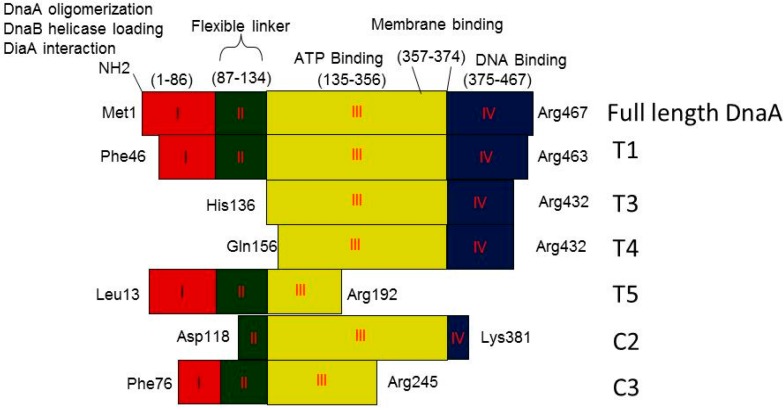
Schematic representation of DnaA domains and Proteolytic fragments obtained with different proteases. Different colors indicate domains I–IV.

Alignment of the primary protein sequences of DnaA protein and proteolytic Fragments obtained with trypsin (T1, T3–T5) and chymotrypsin (C2 and C3) revealed that the protease cleavage sites residing at the amino termini of the fragments lie within domains I, II, and III (Figure 4).

### 2.4. Adenine Nucleotide Mediated Conformational Changes in DnaA Protein Are Required for Remodeling of Nucleoprotein Complexes at E. coli oriC

During most of the *E. coli* cell cycle, within *oriC*, DnaA is bound only to the high affinity sites and the recruitment of additional DnaA prior to initiation of replication results in the formation of replication-efficient open complexes [36]. While it is not known which nucleotide form of DnaA is used in the bORC *in vivo*, *in vitro* either ADP-DnaA or ATP-DnaA are proficient in binding high affinity sites [26,28,29]. Just prior to initiation of chromosome replication, DnaA bound to high affinity sites recruit additional DnaA molecules to occupy the remaining low affinity sites in *oriC* [32,33,37], and mediates origin unwinding to complete pre-RC assembly [29]. *In vitro*, the complete occupation of low affinity sites has been observed only with ATP-DnaA in the presence of 5 mM ATP [29].

The limited proteolysis results, describe above, showed that ATP-DnaA in the presence of ATP, and ADP-DnaA in the presence of ADP have different conformations in the amino half of the protein, which is also the region known to be required for cooperative binding among DnaA molecules [6,7]. To determine if the ATP-specific conformation was required for DnaA recruitment and extension to the low affinity sites in bORC to pre-RC conversion, we used DMS footprinting to examine DnaA binding to form the two complexes under a variety of adenine nucleotide conditions. 

**Figure 5:**
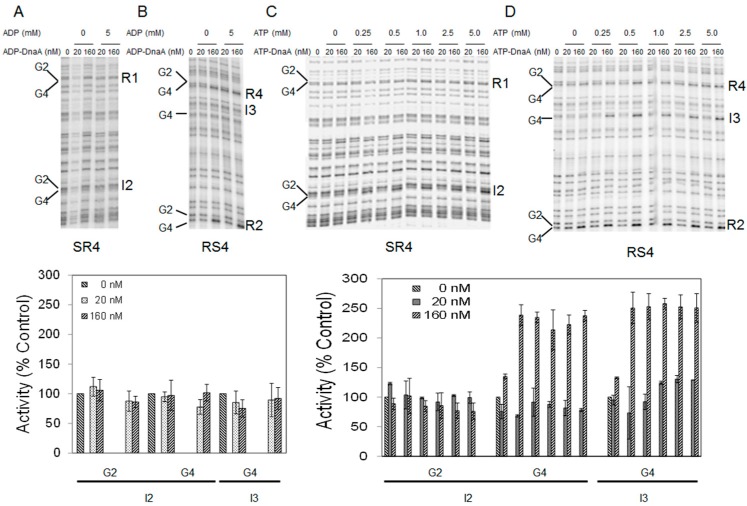
Adenine nucleotide-induced conformational changes are required for pre-RC assembly at *E. coli* chromosomal origin. (**Top**) ADP-DnaA or ATP-DnaA at 20 and 160 nM were incubated with *oriC* DNA in the absence or presence of 5 mM ADP and absence or presence of 0.25, 0.5, 1.0, 2.5 and 5 mM ATP, respectively and modifications to DMS footprint patterns caused by bound protein were assessed. Sites R1, I2, R4, I3, R2 and the guanines at position 2 and 4 within each site are marked. Primer extension was carried out using primer SR4 (**A**,**C**) or primer RS4 (**B**,**D**) [29]. Note: I3 lacks a guanine at position 2; (**Bottom**) Band intensities at positions G2 and G4 for low affinity sites I2 and I3 are plotted relative to the intensity of the corresponding band in the no protein (0 nM) control *Lane*. Inclusion of ADP or ATP is indicated. Error bars denote standard deviation from three independent experiments.

When DnaA binds to the recognition site 5′-TGTGGATAA (or one of the variations of this sequence), methylation of the G4 residue by DMS is enhanced, while methylation of the G2 residue (if present) is repressed (Figure 5A–D). As has been reported previously [26,28,29], incubation of supercoiled *oriC* plasmid with 20 nM ATP-DnaA and 5 mM ATP leads to binding of the high affinity sites (Figure S3, see also the bar graph), while incubation with 160 nM ATP-DnaA and 5 mM ATP results in increased binding of high affinity sites (Figure 5C,D and Figure S3, see also the bar graph) [26,28,29] as well as occupation of low affinity sites in *oriC* (Figure 5C,D, see also bar graph) [26,28,29]. In contrast, ADP-DnaA, in the presence of 0 or 5 mM ADP, was capable of binding only high affinity sites, even at the higher (160 nM) DnaA concentration (Figure 5A,B and Figure S3, see also bar graph) [26,28,29]. These data confirm previous studies [26,28,29] and indicate that either nucleotide form of DnaA is capable of binding high affinity sites in *oriC* and forming the bORC, and extend the findings to demonstrate that bORC formation is not dependent on any added adenine nucleotide.

Although bORC formation does not distinguish between ATP-DnaA or ADP-DnaA, (Figure S3) [26,28,29], it was of interest to determine whether the conversion of bORC to pre-RC required similar ATP concentration as that needed to induce the above described nucleotide-dependent conformational changes. Interestingly, when *oriC* plasmids were incubated with 160 nM ATP-DnaA, only weak binding to low affinity sites could be observed, which became more pronounced when the ATP concentration was increased from 0.25 to 5 mM (Figure 5C,D, see also the bar graph). These results indicate that the amount of ATP that induces conformational changes coincides with the level required for conversion of a bORC to a pre-RC, suggesting the nucleotide-dependent conformational changes are needed for the conversion.

**Figure 6:**
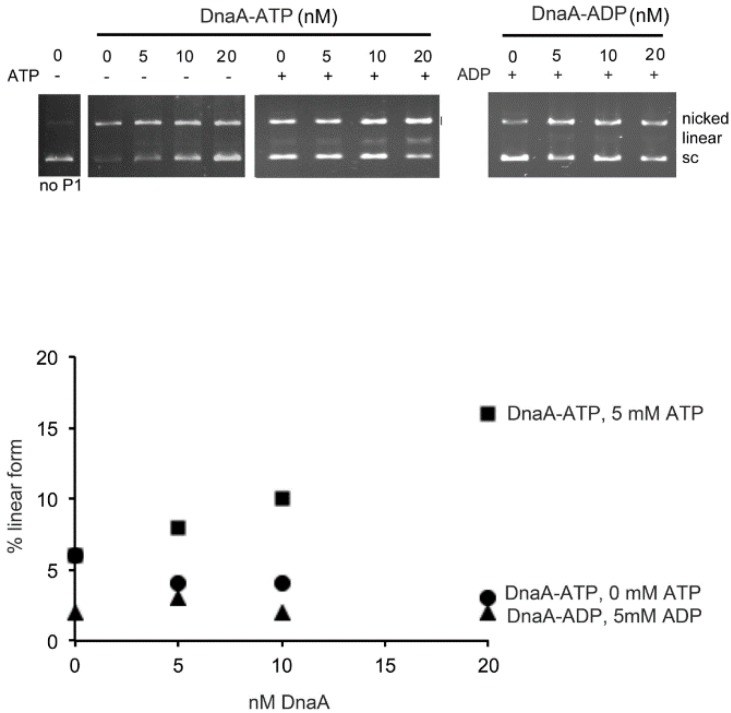
ATP-DnaA requires additional ATP mix to unwind *oriC*. (**Top**) supercoiled *oriC* plasmid was incubated with the indicated concentrations of ATP-DnaA or ADP-DnaA in the absence or presence of 5 mM ATP or 5 mM ADP. After P1 endonuclease digestion, DNA species were resolved by electrophoresis. The supercoiled (sc), nicked, and linear forms are marked; (**Bottom**) the linear form generated by P1 digestion in the presence or absence of 5 mM ATP and 5 mM ADP respectively were quantitated.

The conversion of bORC to pre-RC is coincident with strand separation in the DNA unwinding element (DUE), which consists of the 13-mer repeat region [29]. Consistent with this, previous studies have shown that only ATP-DnaA in the presence of 5 mM ATP, results in full occupation of low affinity sites and effectively unwinds the DNA within the DUE [29]. To verify that a conformational change in ATP-DnaA induced by 5 mM ATP is required for open complex formation, supercoiled *oriC* plasmids were incubated with ATP-DnaA or ADP-DnaA in the absence or presence of 5 mM ATP or 5 mM ADP respectively, followed by treatment with P1 endonuclease. In the absence of P1 endonuclease digestion, only supercoiled DNA is seen (Figure 6, *Lane* 1), while P1 in the absence of DnaA, shows some nicking activity, but no linear forms are generated by two opposing nicks (Figure 6, *Lane* 2), suggesting that the cuts are not localized to any specific region of the plasmid. Increasing amounts of ATP-DnaA in absence of additional ATP are feeble at producing any linear product (Figure 6, *Lane* 3–5), suggesting that localized unwinding in the DUE, which would be susceptible to opposing nicking activity by P1, did not occur. However, as expected, only ATP-DnaA and not ADP-DnaA in the presence of 5 mM ATP or ADP, respectively, generated linear DNA (Figure 6, *cf. Lanes* 6–9 and 10–13), indicating that these conditions are needed for open complex formation. Taken together, our results show that the concentration of ATP required for bORC to pre-RC conversion and duplex melting closely coincides with the amount needed to induce conformational changes, as identified by proteolytic digestion.

## 3. Discussion

ADP and ATP bound to the initiator protein DnaA controls its activity to initiate replication at the *E. coli* chromosomal origin [1,2,3] and regulate transcription of several genes [38,39], including its own [40]. X-ray crystallographic structures of truncated ADP-DnaA and ATP-DnaA protein from the thermophilic bacterium *A. aeolicus* exhibit significant conformational differences between the two nucleotide forms [15,16]. These differences are suggested to be crucial for the oligomerization of ATP-DnaA at *oriC* [16].

Here, molecular docking of ADP and ATP to the homology-modeled structure of full length *E. coli* DnaA, using the *A. aeolicus* DnaA structure as a template, suggests subtle differences in the orientation of the amino acid residues present in the vicinity of nucleotide binding pocket. Interestingly, we saw that ATP-DnaA in the presence of several micromolar to millimolar levels of ATP (comparable to those present at physiological levels in bacteria, approximately 1–3 mM) had different susceptibility for trypsin and chymotrypsin proteases, when compared to ADP-DnaA (physiological levels approximately 0.25 mM, or 10 folds less than ATP).

Digestion of ADP-DnaA and ATP-DnaA with trypsin and chymotrypsin in the presence of approximately physiological levels of ATP reveal nucleotide-dependent conformational changes within DnaA domains I–III (Table 1 and Figure 4). Considering, that these regions are respectively present either in or flanking domain II, which is predicted to be a flexible loop and involved in alignment of domain I with domains III and IV, leads us to a question whether it is possible that ATP or ADP present in the nucleotide pocket of DnaA protein might differentially affect the structure of domain II and thereby influence the initiation activity of DnaA. Doing so would be consistent with the previous observations that a certain minimal domain II is required for the viability of *E. coli* [11].

Although cellular levels of nucleotide and DnaA protein usually remain relatively constant, changes have been reported in the levels of replication inefficient ADP-DnaA and replication proficient ATP-DnaA during the cell cycle [41]. *In vivo*, proper cellular levels of acidic phospholipids such as phosphatidylglycerol (PG) and cardiolipin (CL) are linked to cell growth [42,43] and chromosomal replication [23,44,45]. *In vitro*, PG and CL [46,47] along with DnaA reactivating sequences, DARS [48], have been suggested to catalyze the release of adenine nucleotides bound to DnaA protein. In contrast, mechanisms such as regulatory inactivation of DnaA (RIDA) [49] and presence of *datA* sequences present within chromosomal DNA [50] regulate conversion of ATP-DnaA to ADP-DnaA. Combined, these mechanisms help to maintain the appropriate balances between the two nucleotide forms of the initiator protein.

It is interesting that in *in vitro*, while sub-micromolar levels of ATP or ADP are enough to generate a nucleotide form of DnaA (*K*_D_ of 0.03 and 0.1 µM for ATP and ADP respectively) [51], ATP at several micromolar to millimolar is required to convert bORC to replication proficient open complexes [51,52]. Of note, our study here shows that a similar amount of adenine nucleotide is needed to induce detectable conformational differences in ADP-DnaA *versus* ATP-DnaA, and it correlates to the level of ATP required for the conversion of bORC to pre-RC and subsequent opening of duplex DNA. The possibility that the several micromolar to millimolar ATP concentration might be required for saturating more than one binding site present on DnaA protein, a function specifically required for the conversion of bORC to pre-RC cannot be excluded and the mechanistic detail on these studies needs to be further elaborated.

## 4. Experimental Section

### 4.1. Reagents and Strains

Bacterial growth media were purchased from Difco Laboratories (Franklin Lakes, NJ, USA). DNA purification plasmid midi kit and Nickel nitrilotriacetic acid (Ni^2+^-NTA) agarose matrix were obtained from Qiagen Inc. (Hilden, Germany) Coomassie stain reagents were purchased from Pierce (Rockford, IL, USA) and BioRad (Hercules, CA, USA). Trypsin protease was from GE Biosciences (Pittsburg, PA, USA). Chymotrypsin, and endonuclease P1 used in the study were purchased from Sigma (St. Louis, MO, USA). Sequa Gel solutions to prepare sequencing gels were procured from National Diagnostics (Atlanta, GA, USA). Radioactive (γ-_32_P)-ATP (3000 Ci/mmol) for the labeling of primers used in foot-printing assay was obtained from Perkin Elmer (Waltham, MA, USA). Oligonucleotides used in this study were synthesized by Integrated DNA Technologies (Coralville, IA, USA). Plasmids pZL411 [53], pBS*oriC* and pOC170 [29] used in this study had been previously described.

### 4.2. Expression and Purification of Recombinant DnaA Protein

Recombinant DnaA protein tagged with (10×)-histidine amino acid residues at the N-terminal was expressed and purified as previously described [29,53]. Briefly, cells were grown in Luria-broth to an optical density (OD600) of 0.8, induced for expression of DnaA with IPTG (1 mM) for 90 min, harvested by centrifugation and re-suspended in 20 mM Na_2_PO_4_ (pH 7.8), 0.5 M NaCl, and 5 mM imidazole. Protein was purified in HD buffer (50 mM PIPES (piperazine-*N*,*N*′-bis(2-ethanesulfonic acid) pH 6.8, 0.2 M ammonium sulfate, 10 mM magnesium acetate, 20% sucrose, 2 mM DTT and 0.1 mM EDTA) containing 1 M imidazole [29,53]. The ability of the purified histidine-tagged DnaA to support *in vitro* DNA replication in a crude cell-free extract and its ability to bind ATP were determined to assess the activity of purified DnaA protein, as described elsewhere [53]. To prepare the nucleotide forms of ADP-DnaA or ATP-DnaA, the protein was incubated with 1 μM of ADP or ATP (unless mentioned otherwise), as described previously [29,53].

### 4.3. Bioinformatics and Structural Analysis

The protein sequence information was obtained from the Uniprot database (www.uniprot.org). Domain information was obtained from the pfam database (http://pfam.sanger.ac.uk/). The structural information for the available homologous structures was obtained from the Protein Data Bank (www.rcsb.org). Topological information and interacting residues were obtained from PDBSum (http://www.ebi.ac.uk/pdbsum/) database.

### 4.4. Homology Modeling

A structure-guided alignment of the homologous sequences and structures were performed using Cn3d tool implemented within the CDTree (http://www.ncbi.nlm.nih.gov/Structure/cdtree/cdtree.shtml) tool to pick the best structure as a template for modeling. The structure of truncated DnaA from *A. aeolicus* (PDB-ID 1L8Q) was used as the template for modeling. Homology modeling was done using the Swiss-pdb viewer. The model was refined and minimized using the GROMOS986 potentials implemented in Swiss-pdb viewer. The model that had the least energy and that satisfied geometrical constraints as evident from Ramachandran plot was chosen for further docking studies. Docking of ATP or ADP to the entire energy minimized structure structure was done using Autodock Vina in the flexible docking mode (http://vina.scripps.edu/index.html). DnaA belonging to the superfamily of P-loop ATPases and family of extended AAA+ ATPase domain was used to select the best representative for initial docking.

### 4.5. Protease Digestion

ADP-DnaA or ATP-DnaA protein (1.5 μM) in HD buffer, were subjected to proteolytic digestion by trypsin (TPCK-treated) or chymotrypsin at a molar ratio of 4:1 (DnaA: protease) in the absence or presence of additional ADP and ATP (Mg^2+^ adjusted to 6.5 mM). The molar ratios were calculated considering the molecular mass of 10X-His-DnaA protein as ~53.4 kDa, trypsin as 23.3 kDa and chymotrypsin as 25.6 kDa Briefly, each reaction contains 2.34 µg of DnaA protein and 260 ng of protease in a reaction volume of 30 µL (weight ratio of DnaA:protease is 9:1). Proteolysis was allowed to proceed for 30 min at 30 °C. Each reaction was terminated by the addition of phenylmethanesulfonyl fluoride (PMSF) to a final concentration of 5 mM [54]. Protein fragments were resolved by electrophoresis through 15% SDS-polyacrylamide gels and visualized by Coomassie staining. Protein bands intensities were quantified using image J software (available at http://rsb.info.nih.gov/ij/).

### 4.6. Liquid Chromatography Mass Spectroscopy

Protein bands of interest were manually excised, dehydrated and trypsin-digested at 37 °C for 16–20 h. Separation of protein fragments was performed using Aquity UPLC BEH 130 C18 column (75 μm × 150 mm, 1.7 μm), (from Waters, Milford, MA, USA). The sample manager was thermostated at 4 °C and the column temperature was set at 40 °C. Eluant A was an aqueous solution of 0.1% formic acid and 2% acetonitrile. Eluant B was 0.1% formic acid in acetonitrile. Mass spectra were acquired using a nano-ESI-LC-MS/MS QStar Elite (ABSCIEX, Framingham, MA, USA). Each cycle consisted of a full scan from *m*/*z* 400–1800 and three information dependent acquisitions (IDAs) from *m*/*z* 100–1600 for the top three ions that exhibited the highest signal intensity, met minimum 5 counts and also carry 2–5 charges. Former target ions were excluded for 20 s. Mass tolerance was set to 100 ppm. Mass spectra from three injections of each sample were pooled together to perform search against the SWISS-PROT databases using ProteinPilot software 4.0 and Paragon Algorithm. Gel-based ID and biological modifications were included in the search parameters.

### 4.7. DMS Footprinting

*In vitro* dimethylsufate (DMS) footprinting was carried out as described previously [26,29]. Briefly, ADP-DnaA or ATP-DnaA proteins were mixed with *oriC* DNA (0.5 μg) and incubated in the presence of different amounts of ADP and ATP for 7 min at 38 °C. To assess DNA binding, changes in DNA methylation patterns were examined by treatment with dimethylsulfate (6 μL of 1.4% *v*/*v*) for 5 min and the reactions were stopped with a solution (200 μL) of 3 M Ammonium acetate, 1 M 2-mecaptoethanol, 250 μg/mL tRNA, 20 mM EDTA. The DMS-treated DNA samples were incubated with piperidine for 30 min at 95 °C, and the piperidine generated fragments were subsequently isolated using Micro Bio-Spin 6 chromatography columns (Bio-Rad, Hercules, CA, USA). Binding of ADP-DnaA or ATP-DnaA protein to specific DnaA binding sites was examined by primer extension using radiolabeled primers. For the primer extension reactions samples were processed, air-dried, and dissolved in gel loading buffer as described previously [26,29]. The radiolabeled extension products resolved through 6% acrylamide sequencing gels were detected with a Molecular Dynamics STORM 840 imager and analyzed using Bio-Rad QUANTITY ONE software. 

### 4.8. P1 Endonuclease Digestion

P1 endonuclease digestions were performed as described previously [29]. Briefly, supercoiled *oriC* plasmid (10 fmol; pOC170) in 20 μL of 40 mM HEPES-KOH, pH 7.6, 8 mM MgCl_2_, 30% glycerol and 0.32 mg/mL BSA in absence or presence of 5 mM ATP or ADP (as indicated in Figure 6) was incubated with increasing concentrations of ATP-DnaA or ADP-DnaA for 5 min at 38 °C. P1 endonuclease (0.6 units) was added for 20 s and the reaction was subsequently stopped by the addition of stop solution (2% SDS and 50 mM EDTA). Samples were heated (65 °C) for 5 min and DNA species were resolved by electrophoresis through 1% agarose gel following its staining with ethidium bromide. Quantitation of the relative amounts of each form was done using Bio-Rad QUANTITY ONE software.

## Supplementary Materials

Supplementary materials can be found at http://www.mdpi.com/1422-0067/16/11/26064/s1.
