# Supplementary material for: Nucleotide-Induced Conformational Changes in Escherichia coli DnaA Protein Are Required for Bacterial ORC to Pre-RC Conversion at the Chromosomal Origin
Source: Int J Mol Sci. 2015 Nov 24;16(11):27897–911. doi: 10.3390/ijms161126064 (PMC4661922; doi:10.3390/ijms161126064)
Supplement: Supplementary file 1 [file ijms-16-26064-s001.pdf]

Supplementary Materials: Nucleotide-Induced Conformational Changes in *Escherichia coli* DnaA Protein are Required for Bacterial ORC to Pre-RC Conversion at the Chromosomal Origin

Rahul Saxena , Sona Vasudevan, Digvijay Patil, Norah Ashoura, Julia E. Grimwade and Elliott Crooke

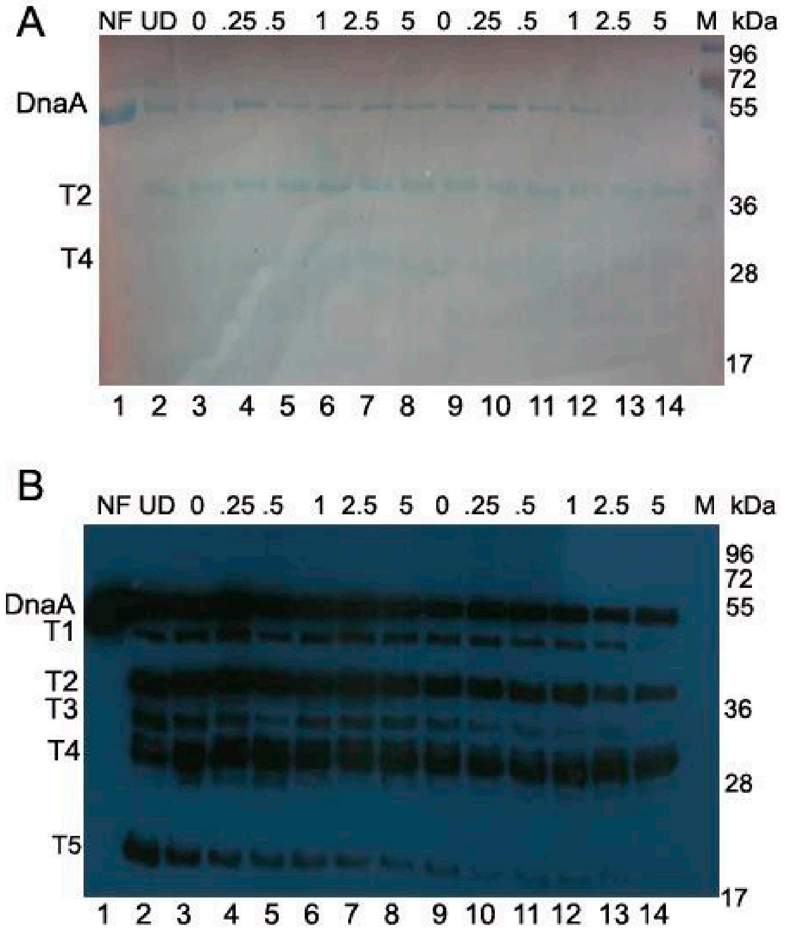

Figure S 1. ADP-DnaA and ATP-DnaA confers different resistance to trypsin protease. (**A**,**B**) Nucleotide free, ADP and ATP-DnaA protein (1.5 μM) were subjected to proteolysis by trypsin (**A**) (Protein: Protease molar ratio of 4.0) for 30 min at 30 °C. *Lane 1*, Undigested (UD) nucleotide free DnaA; *Lane 2*, Nucleotide free DnaA (NF) treated with trypsin (**A**); *Lane 3*, ADP-DnaA treated with trypsin (**A**); *Lanes 4–8*, ADP-DnaA treated with trypsin (**A**) in the presence of 0.25, 0.5, 1.0, 2.5 and 5 mM ADP; *Lane 9*, ATP-DnaA treated with trypsin (**A**); *Lanes 10–14*, ATP-DnaA treated with trypsin (**A**) in the presence of 0.25, 0.5, 1.0, 2.5 and 5 mM ATP. Protein fragments were resolved by electrophoresis through 15% SDS-polyacrylamide gels and visualized by Ponceau stain (**A**) and immunoblotting with anti-DnaA antibodies (**B**).

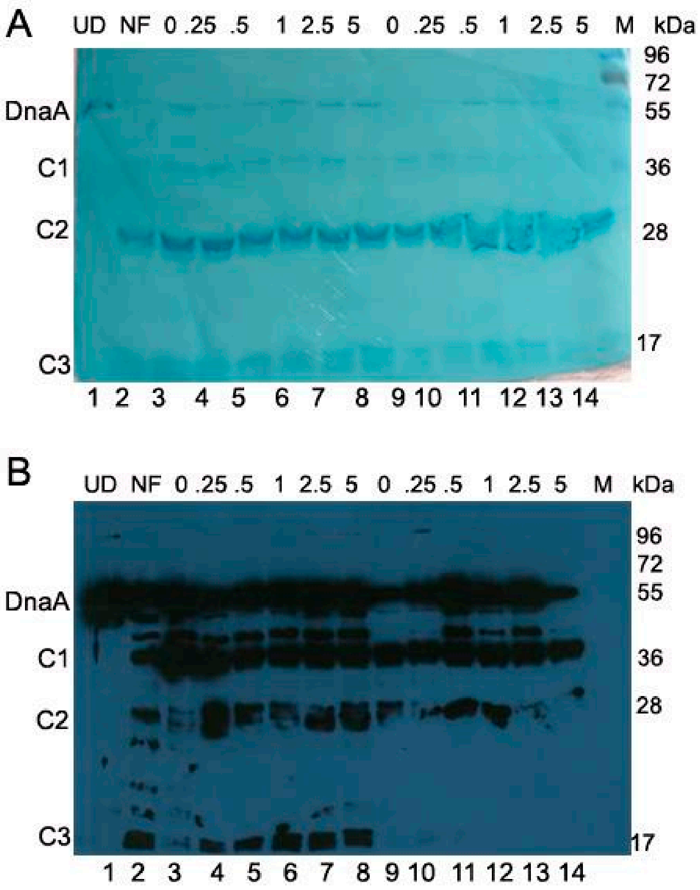

Figure S 2. ADP-DnaA and ATP-DnaA confers different resistance to chymotrypsin protease. (**A**,**B**) Nucleotide free, ADP and ATP-DnaA protein (1.5 μM) were subjected to proteolysis by chymotrypsin (**A**) (protein: protease molar ratio of 4.0) for 30 minutes at 30 °C. *Lane 1*, Undigested (UD) nucleotide free DnaA; *Lane 2*, Nucleotide free DnaA (NF) treated with chymotrypsin (**A**); *Lane 3*, ADP-DnaA treated with chymotrypsin (**A**); *Lanes 4–8*, ADP-DnaA treated with chymotrypsin (**A**) in the presence of 0.25, 0.5, 1.0, 2.5 and 5 mM ADP; *Lane 9*, ATP-DnaA treated with chymotrypsin (**A**); *Lanes 10–14*, ATP-DnaA treated with chymotrypsin (**A**) in the presence of 0.25, 0.5, 1.0, 2.5 and 5 mM ATP. Protein fragments were resolved by electrophoresis through 15% SDS-polyacrylamide gels and visualized by Ponceau stain (**A**) and immunoblotting with anti-DnaA antibodies (**B**).

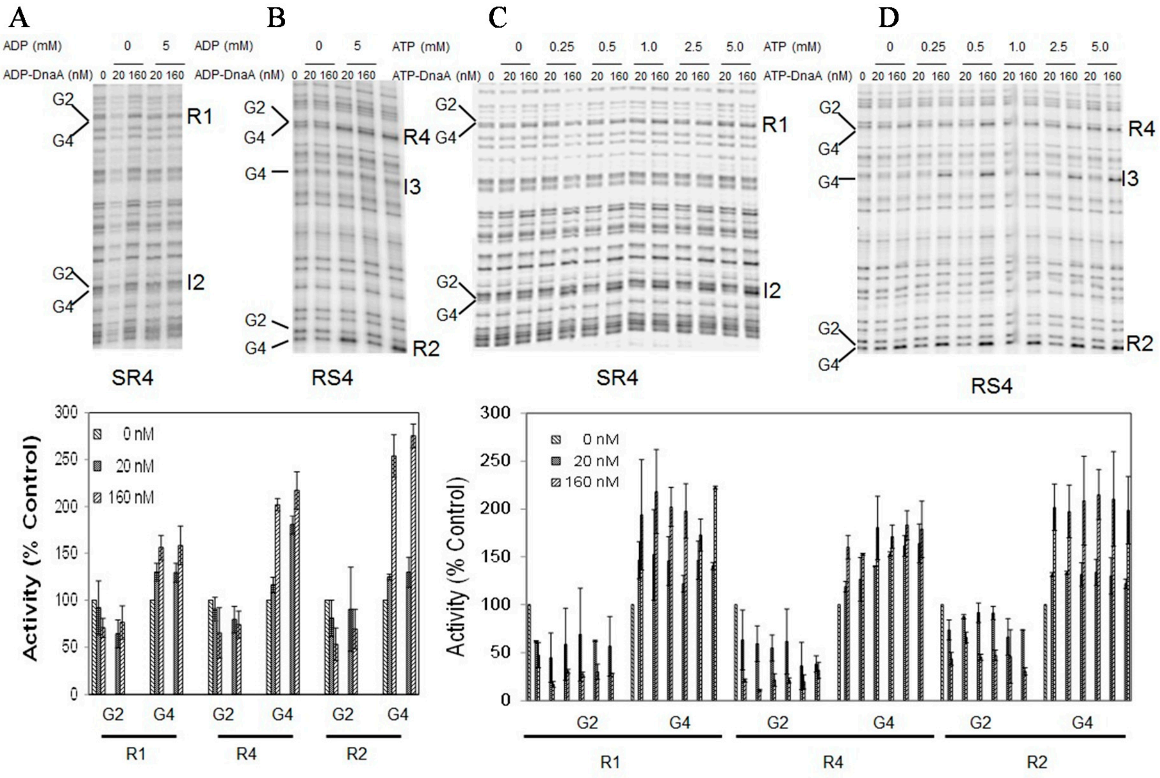

Figure S3. DMS footprint of high affinity sites by ADP and ATP forms of DnaA. (**Top**) ADP-DnaA or ATP-DnaA at 20 and 160 nM were incubated with oriC DNA in the absence or presence of 5 mM ADP and absence or presence of 0.25, 0.5, 1.0, 2.5 and 5 mM ATP, respectively and modifications to DMS footprint patterns caused by bound protein were assessed. Sites R1, I2, R4, I3, R2 and the guanines at position 2 and 4 within each site are marked. Primer extension was carried out using primer SR4 (**A**,**C**) or primer RS4 (**B**,**D**) [29]; (**Bottom**) Band intensities at positions G2 and G4 for R1, R2, R4 are plotted relative to the intensity of the corresponding band in the no protein (0 nM) control lane. Error bars denote standard deviation from three independent experiments.
